# Multi-fidelity modelling of shark skin denticle flows: insights into drag generation mechanisms

**DOI:** 10.1098/rsos.220684

**Published:** 2023-02-01

**Authors:** C. J. Lloyd, K. Mittal, S. Dutta, R. M. Dorrell, J. Peakall, G. M. Keevil, A. D. Burns

**Affiliations:** ^1^ Energy and Environment Institute, University of Hull, Hull, UK; ^2^ Mechanical Engineering, University of Illinois Urbana-Champaign, Urbana, IL, USA; ^3^ Mechanical and Aerospace Engineering, Utah State University, Logan, UT, USA; ^4^ Earth and Environment, University of Leeds, Leeds, West Yorkshire, UK; ^5^ School of Chemical And Process Engineering, University of Leeds, Leeds, West Yorkshire, UK

**Keywords:** drag reduction, shark skin, dermal denticles

## Abstract

We investigate the flow over smooth (non-ribletted) shark skin denticles in an open-channel flow using direct numerical simulation (DNS) and two Reynolds averaged Navier–Stokes (RANS) closures. Large peaks in pressure and viscous drag are observed at the denticle crown edges, where they are exposed to high-speed fluid which penetrates between individual denticles, increasing shear and turbulence. Strong lift forces lead to a positive spanwise torque acting on individual denticles, potentially encouraging bristling if the denticles were not fixed. However, DNS predicts that denticles ultimately increase drag by 58% compared to a flat plate. Good predictions of drag distributions are obtained by RANS models, although an underestimation of turbulent kinetic energy production leads to an underprediction of drag. Nevertheless, RANS methods correctly predict trends in the drag data and the regions contributing most to viscous and pressure drag. Subsequently, RANS models are used to investigate the dependence of drag on the flow blockage ratio (boundary layer to roughness height ratio), finding that the drag increase due to denticles is halved when the blockage ratio *δ*/*h* is increased from 14 to 45. Our results provide an integrated understanding of the drag over non-ribletted denticles, enabling existing diverse drag data to be explained.

## 1. Introduction

For decades scientists and engineers have been fascinated by the skin of sharks, composed of sub-millimetre scale dermal denticles. These complex structures protrude from a flexible epidermis and vary considerably in shape depending on the shark species and location on the body [1–4]. There is a particular interest in the hydrodynamics of shark scales; studies have shown they can reduce skin friction drag (e.g. [5–8]) and enhance hydrodynamic performance in separating flows (e.g. [9,10]). The most extensive datasets focus on the riblet features typically present on the denticles of fast-swimming sharks, which protrude from the denticle crown. Theoretical, experimental and numerical studies have shown that idealized two-dimensional riblets can reduce drag by up to 10% by suppressing spanwise motion in the near-wall region which could otherwise lead to increased mixing in the boundary layer [6,11–14]. The performance of riblets scales with their dimensionless spacing, $s^{+}=s/\delta_{\nu }$,^1^ where the dimensional spacing *s* is normalized by the viscous wall-unit length $\delta_{\nu }=\nu /u_{\tau }$, where *ν* is the kinematic viscosity and $u_{\tau }$ is the friction velocity, related to the wall shear stress *τ*_*w*_ and density *ρ* by $u_{\tau }^{2}=\tau_{w}/\rho$ [15]. When *s*^+^ is O(1) riblets lie in the viscous sub-layer and drag reduction scales linearly with *s*^+^. As riblets grow larger they protrude into the turbulent regions of the boundary layer and performance degrades; they are no longer able to restrict spanwise motion and instead increase turbulent mixing [15].

However, there is substantial disagreement between comparable studies of the flow over ‘real’ three-dimensional shark scales; some studies report poor hydrodynamic performance of denticles compared to a flat plate [16–18], some report similar performance to idealized riblets [5,8], and some report better performance than longitudinal riblets, where skin friction drag is reduced by up to 30% [7,19]. The vast range of contradictory results presented in the literature are in part due to differences in denticle geometries and spacings, arising from differences in denticle samples and manufacturing techniques [18]. As of yet a comprehensive study on the effects of denticle geometry and spacing on skin friction has not been carried out. A further key issue is that near-denticle flow is rarely quantified [17,18], and so mechanisms responsible for drag performance are unknown.

Numerical methodology offers an alternative to experiments and can capture the flow physics around individual, and arrays of, denticles. To date only Zhang *et al.* [20] and Boomsma & Sotiropoulos [17] have carried out roughness-resolving experimentation using computational fluid dynamics (CFD). Boomsma & Sotiropoulos [17] adopted direct numerical simulation (DNS) to model the flow around an array of Mako scales. DNS fully resolves the flow field and thus does not require models to account for turbulence, making it an attractive methodology for identifying drag reducing mechanisms (see e.g. [11]). However, its computational expense limits its use to low Reynolds numbers. This limitation is a possible cause of the poor agreement between the DNS of Boomsma & Sotiropoulos [17] and the experiments of Wen *et al.* [8]; the simulations of Boomsma & Sotiropoulos [17] predict a drag increase of 50% compared to a flat plate, while the experiments of Wen *et al.* [8] measured a drag increase of approximately 5%, both for the same denticle geometry with *s*^+^ ≈ 16. By contrast, methodology based on the Reynolds averaged Navier–Stokes (RANS) equations are not so limited by Reynolds number, but require validation against benchmark data. To date, only Zhang *et al.* [20] have carried out RANS simulations on resolved shark scales, but it is unclear how well RANS methodology is able to predict the flow over geometrically resolved rough surfaces (i.e without using roughness wall functions, which have been used to simulate the flow over full shark bodies by Díez *et al.* [21] and Zhang *et al.* [22]). However, the method may provide valuable insight into the fluid dynamics of shark scales by simulating more realistic flow scenarios when compared to DNS. Its low cost also enables vast parameter studies on denticle geometry.

In this paper, we investigate the performance of rigid, smooth denticles in a low Reynolds number periodic open channel flow. Our work focuses on denticle performance in attached canonical wall-bounded flows, rather than more complex separating flows where shark scales may offer other advantages [23], for example, by passive bristling enabled by the flexible epidermis [10]. Smooth denticles, without ridges/riblets present on the denticle crown, are chosen to enable comparison against the experiments of Lloyd *et al.* [18], and to act as a baseline case for future studies investigating the influence of denticle geometry on attached boundary layer dynamics, and for examining the interaction between denticles and riblets [18]. A DNS is performed, adopting a novel high-fidelity Schwarz-spectral element method (SSEM), where the fluid domain is discretized using overlapping body-fitted grids. In addition, two RANS models are validated against DNS data; one based on the *k* − *τ* model of Kalitzin *et al.* [24], and another based on the EB-SSG Reynolds Stress model of Manceau [25]. Upon finding a drag increase, relative to a flat plate, we assess flow characteristics that lead to such high drag, identifying high levels of shear, turbulent kinetic energy (TKE) production and a high momentum pathway between individual denticles. In addition, we investigate the hydrodynamic lift forces acting on the denticles, and make comparisons to the simulations of Boomsma & Sotiropoulos [17] and the experiments of Lloyd *et al.* [18]. Finally, we investigate the dependence of drag on the flow blockage ratio, equal to the boundary layer thickness normalized by the roughness height, using RANS simulations. Results show vast improvements in hydrodynamic performance when the blockage ratio is increased, potentially explaining the vast differences in drag estimations from previous studies.

We conclude by discussing the open questions regarding hydrodynamic performance of shark scales in canonical boundary layer flows, and how they may be addressed in future studies.

## 2. Methodology

### 2.1. Denticle and domain geometry

**Figure 1.**
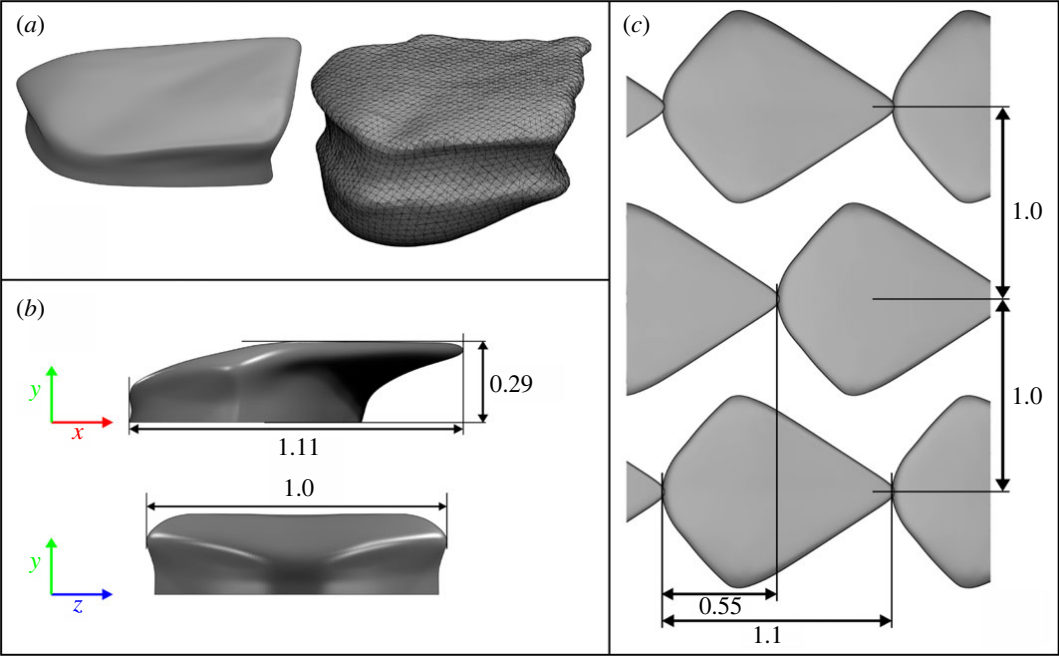
CAD design and dimensions, scaled by the denticle width *w*. (*a*) The smoothed three-dimensional CAD model (left) compared to the original ≈1 mm width *Poracanthodes sp.* sample (right). The main denticle dimensions are presented in (*b*) and array dimensions are given in (*c*).

This work adopts a denticle CAD model used in the experimental study of Lloyd *et al.* [18], which can be observed in figure 1, with dimensions scaled by the denticle width, *w*. The denticle is based on a *Poracanthodes sp.* sample, an early fossil ancestor of sharks, chosen due to its simplicity and similarity with other denticles common in hydrodynamic studies [8,16], minus the riblets on the denticle crown. Like modern sharks, the *Poracanthodes sp.* denticle has an overhanging crown, a sharp trailing edge and a slightly thinner neck region below which the denticle embeds into the dermis [4]. Using Blender [26] CAD software the fossil sample was made symmetrical and smoothed along the trailing edge in order to remove imperfections. The model is also clipped at the base of the neck region such that only material exposed to water is simulated. The resulting denticle modelled in our simulation has the dimensions *L*_*x*_ = 1.1*w*, *L*_*y*_ = 0.29*w* and *L*_*z*_ = *w*, where *w* is the denticle width.

**Figure 2.**
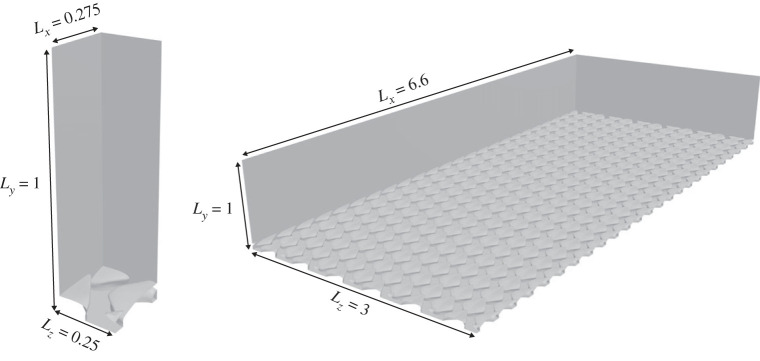
RANS (left) and DNS (right) fluid domains for open-channel flow simulation with smooth denticles on the wall. *x* is the streamwise direction, *y* is the vertical direction and *z* is the spanwise direction.

Figure 2 shows the fluid domains for the RANS and DNS studies. The RANS domain contains a single denticle, symmetric in the spanwise (*z*) direction and periodic in the streamwise direction (*x*). The DNS domain is doubly periodic (in *x* and *z*) and contains 24 denticles in the *x*-direction and 12 denticles in the *z*-direction. A fully developed open-channel flow, with a symmetry condition imposed at the upper boundary, is simulated over the denticles with a friction Reynolds number $Re_{\tau }=u_{\tau }\delta /\nu \approx 180$, enforced by imposing a bulk velocity/flow rate. Here, *u*_*τ*_ is the friction velocity, *δ* is the open-channel height (measured from the base of the denticles) and *ν* is the kinematic viscosity. The denticle width is scaled to *w*/*δ* = 0.25, where *δ* is the channel height, and subsequently *w*^+^ = *wu*_*τ*0_/*ν* ≈ 45, where *u*_τ0_ represents a reference smooth-walled friction velocity, equating to a slightly larger denticle width than that of Boomsma & Sotiropoulos [17] (*w*^+^ ≈ 38.4). This is compensated by a reduced denticle height of *h*^+^ = *hu*_*τ*0_/*ν* ≈ 13.05 where *h* is the denticle height, compared to that of Boomsma & Sotiropoulos [17] (*h*^+^ ≈ 22) whose larger denticle height is partly due to the riblets protruding from the denticle crown. Note that the denticle size is quantified by its inner-scaled width *w*^+^ rather than the inner-scaled riblet spacing *s*^+^ as per typical studies on shark scales and riblets, due to the lack of riblets on the denticle crown. The blockage ratio, defined as the ratio between the boundary layer and roughness heights, of the present simulations is reasonably small at *δ*/*h* ≈ 14, such that the effects of roughness will not be limited to the inner region (y≲0.2δ) of the boundary layer [27]. The implications and effects of this will be discussed in §3.

The resulting RANS domain is *L*_*x*_ × *L*_*y*_ × *L*_*z*_ = 0.275*δ* × *δ* × 0.25*δ* and has 1 denticle, and the DNS domain is *L*_*x*_ × *L*_*y*_ × *L*_*z*_ = 6.6*δ* × *δ* × 3*δ* with a total of 284 denticles (24 denticles in the *x*-direction and 12 denticles in the *z*-direction). The size of the DNS domain is consistent with Boomsma & Sotiropoulos [17].

### 2.2. DNS methodology

We use DNS to solve the dimensionless incompressible Navier–Stokes equations and the continuity equation:

2.1 $$\frac{\partial U_{i}}{\partial t}+U_{j}\frac{\partial U_{i}}{\partial x_{j}}=-\frac{\partial P}{\partial x_{i}}+\frac{1}{Re_{b}}\frac{\partial^{2}U_{i}}{\partial x_{j}\partial x_{j}}+f_{i},$$

and

2.2 $$\frac{\partial U_{i}}{\partial x_{i}}=0,$$

where *U*_*i*_ = (*U*, *V*, *W*)′ is the three-dimensional velocity field, *x*_*i*_ = (*x*, *y*, *z*)′ represents Cartesian space, *P* represents the kinematic pressure and *f*_*i*_ = (*f*_*x*_, 0, 0)′ is an iterative (negative) pressure gradient designed to impose a constant bulk flow rate through the domain. Flow dynamics are controlled by the bulk Reynolds number *Re*_*b*_ = *U*_*b*_*δ*/*ν* = 2812.23, where *ν* is the kinematic viscosity, *U*_*b*_ is the bulk velocity and *δ* is the channel height. *f*_*x*_ iteratively controls the bulk flow rate through the domain, and therefore the bulk Reynolds number. The bulk flow rate *Q* is set equal to *U*_*b*_*L*_*x*_*L*_*z*_, where *L*_*x*_*L*_*z*_ is the frontal area of the domain in the absence of the denticles. Note that this leads to a fractionally higher bulk Reynolds number (*Re*_*b*_ = 2900.12 instead of 2812.23) than that imposed in equation (2.1), due to the presence of the denticles which effectively reduces the reference area, and therefore increases *U*_*b*_. We show that the impact of this increase in *Re*_*b*_ on drag characteristics is insignificant, in §3.

The governing equations are discretized using an overlapping SSEM implemented in NEK5000 [28]. The SSEM framework is based on the principles of the overlapping Schwarz (OS) method for solving PDEs on overlapping domains. The key benefit of this approach is a reduced grid resolution necessary for fully resolved simulations, where the near-denticle grid can be discretized using a high resolution body-fitted mesh, and the freestream can be discretized with a lower resolution computational mesh (although still high enough to fully resolve all the important scales of the turbulent flow). SSEM has been successfully adopted to simulate a range of wall-bounded flows (e.g. [29–33]).

In standard SEM, the computational domain is defined as a union of *E* spectral elements. The SEM is a high-order weighted residual method where the basis functions are a tensor product of the *N*th-order Lagrange interpolants on the Gauss–Lobatto–Legendre (GLL) points inside each element. The unsteady governing equations (2.1) and (2.2) are solved in the velocity–pressure form using semi-implicit BDF3/EXT3 timestepping, in which the time derivative is approximated by a third-order backward difference formula (BDF3), the nonlinear terms and forcing term are treated with third-order extrapolation (EXT3), and the viscous and pressure terms are treated implicitly. Please see Mittal *et al.* [34] for a detailed description of the timestepping methodology.

**Figure 3.**
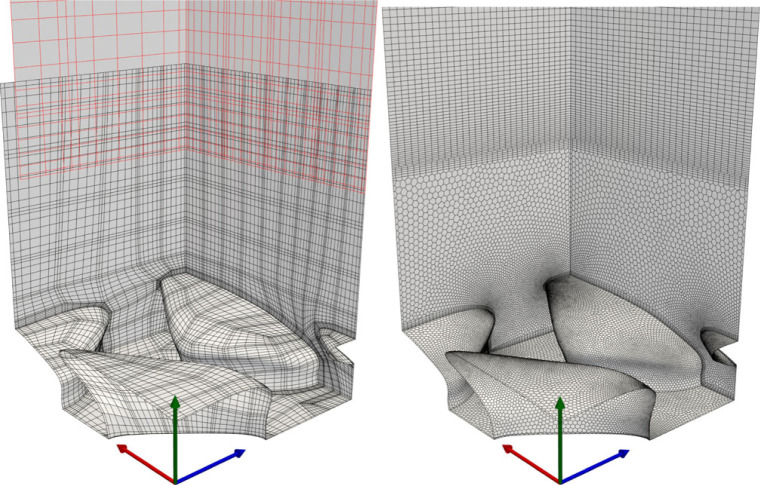
Meshes for DNS (left) and RANS (right) studies. Axes are coloured by *x* (red), *y* (green), *z* (blue).

In the current SSEM-based DNS, the fluid domain is split (vertically) into two sub-domains, each represented by separate overlapping grids: the freestream domain, and the near-denticle domain. The freestream domain is discretized with a uniform hexahedral grid of size *L*_*x*_ × *L*_*z*_ = 6.6 × 3.0, spanning the vertical region *y* ∈ [0.25, 1.0]. The grid resolution is 28 × 8 × 18, with a stretching function adopted in the vertical direction to blend finer-resolution near-denticle cells into the freestream. Cells are also refined (vertically) near the zero-gradient upper boundary at *y* = 1. The lower domain is meshed using ANSYS ICEM 19.2 [35]. A body-fitted hexahedral mesh is created for the small one-denticle periodic section of figure 2, spanning the vertical region *y* ∈ [0.0, 0.33]. The mesh is refined near regions of high curvature, and GLL nodes in each element conform to the denticle CAD model. The small single denticle section is subsequently smoothed using a mesh smoothing algorithm [36,37], reflected in the spanwise (*z*) direction to make it symmetric, and translated in *x* and *z* to create a *L*_*x*_ × *L*_*z*_ = 6.6 × 3.0 periodic sub-domain. The lower sub-domain is meshed using 97 632 elements, and the upper domain contains 4032 elements. Note that in the SEM, each of the elements is further discretized by GLL points. The results presented in this paper use *N* = 7, which results in 8^3^ GLL points inside each element. The overlapping grids are shown with the GLL points in figure 3.

Numerically, the governing equations are solved on the overlapping grids simultaneously, with boundary data exchanged between the two overlapping sections by spectral interpolation and third-order temporal extrapolation. At the end of each timestep, three Schwarz iterations are carried out to ensure consistency in the solution in the overlapping region of the two sub-domains. The order of temporal extrapolation, number of Schwarz iterations and sub-domain grid resolutions were determined through a sensitivity study carried out on a smaller domain of *L*_*x*_ × *L*_*z*_ = 3.3 × 1 (section 9.5 of [38]). Sensitivity of solutions to grid resolution was also assessed using the smaller *L*_*x*_ × *L*_*z*_ = 3.3 × 1 domain. Here, we simulated the flow over denticles using a lower polynomial order of *N* = 6 before increasing to *N* = 7, an effective 60% increase in resolution. Drag calculations varied by 0.2% between the two simulations. Mesh statistics for the upper and lower SSEM domains are presented in table 1, where case DR represents the rough surface DNS. The DNS was run for more than 100 flow-through times with time-averaged statistics collected for the last 75 flow-through times. Additionally, in order to spatially average the results, block-wise averaging is performed to combine the statistics for the 24 × 12 denticles that were replicated from the single block containing one denticle (figure 1).

**Table 1.** Mesh statistics for DNS and RANS simulations. The first letter of the label represents the model type (*D* for DNS; *E* for EB-SSG; *K* for *k* − *τ*), the second letter represents the geometry (*R* for rough, *F* for flat). Upper table are denticle meshes, and lower table are structured meshes. Both the overlapping upper (structured) and lower (denticle) meshes are reported for case DR. Domain lengths *L*_*x*_, *L*_*y*_ and *L*_*z*_ are reported, along with the number of denticles resolved in each direction, *N*_*x*_ and *N*_*z*_. The total number of elements is *E* for the denticle meshes, and the number of elements in each direction is reported for the structured meshes: *E*_*x*_, *E*_*y*_ and *E*_*z*_. Element sizes are also reported, scaled in wall units ($\Delta^{+}=\Delta u_{\tau }/\nu$, where Δ is the element size). For the denticle grids the element sizes are taken as the cube-root of the element volume. Structured grids have their *x*, *y* and *z* element sizes reported. Note that the DNS cases, DR, DF and DFb, are further discretized by order seven polynomials, increasing the effective grid resolution by approximately 7^3^.

denticle meshes								
case	*L*_*x*_	*L*_*y*_	*L*_*z*_	*E*	*N*_*x*_	*N*_*z*_	$\Delta_{\min}^{+}$	$\Delta_{\max}^{+}$
DR lower	6.6	0.33	3.0	97 632	24	12	2.4	14.0
ER	0.275	1.0	0.25	294 663	1	1	0.09	2.5
KR	0.275	1.0	0.25	294 663	1	1	0.1	2.7

structured meshes										
case	*L*_*x*_	*L*_*y*_	*L*_*z*_	*E*_*x*_	*E*_*y*_	*E*_*z*_	$\Delta_{x}^{+}$	$\Delta_{y,\min}^{+}$	$\Delta_{y,\max}^{+}$	$\Delta_{z}^{+}$
DR upper	6.6	0.75	3.0	28	8	18	54.6	0.23	32.4	38.6
DF	6.6	1.0	3.0	28	24	18	43.5	1.1	28.3	30.7
DFb	6.6	1.0	3.0	28	24	18	42.3	1.1	27.6	29.9
EF	—	1.0	—	—	143	—	—	0.1	4.9	—
KF	—	1.0	—	—	143	—	—	0.12	5.1	—

### 2.3. Reynolds averaged Navier–Stokes

The RANS simulations solve the dimensionless Reynolds-averaged momentum and continuity equations:

2.3 $$\frac{\partial {\bar{U}}_{i}}{\partial t}+{\bar{U}}_{j}\frac{\partial {\bar{U}}_{i}}{\partial x_{j}}=-\frac{\partial \bar{P}}{\partial x_{i}}+\frac{1}{Re_{b}}\frac{\partial^{2}{\bar{U}}_{i}}{\partial x_{j}\partial x_{j}}-\frac{\partial \bar{u_{i}u_{j}}}{\partial x_{j}}+f_{i},$$

and

2.4 $$\frac{\partial {\bar{U}}_{i}}{\partial x_{i}}=0,$$

where variables are made dimensionless by the channel height *δ* and the reference bulk velocity, *U*_*b*_, as per the DNS methodology of §2.2. In our RANS framework [39], the bulk Reynolds number is directly imposed by the iterative forcing term *f*_*i*_. In (2.3) and (2.4), overbars represent ensemble averaging with a variable decomposed by its mean and fluctuating component: e.g. $U_{i}={\bar{U}}_{i}+u_{i}$. The Reynolds stresses, $-\bar{u_{i}u_{j}}$ are closed by either the two equation *k* − *τ* turbulence model with low Reynolds number corrections, detailed by Lloyd *et al.* [40] and originally developed by Kalitzin *et al.* [24], or the elliptic-blending (EB) SSG model of Manceau [25]. Both these models are suitable for resolving near-wall flow dynamics, integrating fully through the boundary layer without requiring ‘log-law’ wall functions. They differ in their complexity; the *k* − *τ* model assumes the Reynolds stresses are related to a scalar eddy-viscosity, while the (eight-equation) EB-SSG model solves each component of $\bar{u_{i}u_{j}}$ separately, better approximating near-wall anisotropy. Here, we adopt the low Reynolds number version of the EB-SSG model, detailed by Manceau [25].

The periodic and symmetric single denticle fluid domain of figure 2 is discretized using a body-fitted polyhedral mesh near the denticle surface (0 < *y* < *w*) which blends into a hexahedral mesh in the freestream (*w* < *y* < *δ*). Boundary conditions are periodic in the streamwise direction, symmetric in the spanwise direction, symmetric at the channel height *y* = *δ* and no-slip on the denticle surface. The interface between the periodic faces and the two mesh regions (polyhedral/hexahedral) are non-conformal such that high mesh refinement is required at these boundaries to reduce discretization errors. The streamwise flow rate is maintained via an iterative source term *f*_*i*_ = (*f*_*x*_, 0, 0)′, as per [41]. The RANS simulations impose *Re*_*b*_ = 2812.23, equating to $Re_{\tau }\approx 180$ for a smooth-walled channel. The polyhedral/hexahedral mesh can be observed in figure 3. The mesh is refined near regions of high curvature and contains a total of 294 663 computational cells, detailed in table 1, where cases ER and KR represent the rough flow simulations using EB-SSG and *k* − *τ*, respectively. This mesh resolution was deemed appropriate through a mesh sensitivity study; approximately doubling the total number of cells affects the friction predictions by less than 1%. Note that when compared to the DNS grid in figure 3 and table 1 the RANS meshes have a slightly higher refinement, per denticle. We find the RANS methods have a higher sensitivity to grid size than the DNS due to the non-conformal boundaries. Further, since only a single denticle is simulated for the RANS models there is little extra cost associated with higher resolution simulations.

The RANS equations are discretized and solved using OpenFOAM [39]; a second order upwind scheme is adopted for velocity convective terms, and a second order accurate total variation diminishing (TVD) scheme is adopted for all other variables. Laplacian terms are discretized using standard Gaussian integration, and face gradients are calculated using linear interpolation. Corrections are made to account for non-orthogonality when calculating face fluxes. The SIMPLEC scheme of Van Doormaal & Raithby [42] is adopted to couple pressure and velocity equations, and convergence is determined by monitoring the friction velocity, $u_{\tau }$, which converges with a relative error of 1 × 10^−7^ for all cases.

## 3. Results

Simulation parameters are presented in table 2. In addition to the rough-wall flows, smooth wall flows have been simulated for each technique using the same methodologies described in §2. The smooth wall DNS is carried out using SEM (without an overlapping grid) with a mesh containing 28 × 24 × 18 elements and the same domain dimensions and bulk Reynolds number (2900.56) as the rough-wall DNS. A second reference smooth wall DNS is carried out at the same bulk Reynolds number (2812.23) as the RANS simulations, finding that the drag coefficient varies by less than 1% over the small Reynolds number discrepancy.

**Table 2.** Simulation parameters. The first letter of the label represents the model type (*D* for DNS; *E* for EB-SSG; *K* for *k* − *τ*), the second letter represents the geometry (*R* for rough, *F* for flat). Columns list the simulation Reynolds numbers based on friction velocity ($Re_{\tau }=u_{\tau }\delta /\nu$) and bulk velocity ($Re_{b}=U_{b}\delta /u_{\tau }$), total friction ($C_{f}=F_{T,x}/\frac{1}{2}\rho U_{b}^{2}L_{x}L_{z}$) and lift coefficients ($C_{l}=F_{T,y}/\frac{1}{2}\rho U_{b}^{2}L_{x}L_{z}$), and dimensionless denticle width (*w*^+^ = *wu*_*τ*0_/*ν*). *C*_*f*0_ represents the reference smooth-walled data, *F*_*T*,*x*_ is the total streamwise drag force, *F*_*ν*,*x*_ is the total streamwise viscous force and *F*_*p*,*x*_ is the total streamwise pressure force.

label	$Re_{\tau }$	*Re*_*b*_	*C*_*f*_	*C*_*l*_	*w*^+^	*y*_0_	$k_{s}^{+}$	*C*_*f*_/*C*_*f*0_	*C*_*f*_/*C*_*l*_	*F*_*ν*,*x*_/*F*_*T*,*x*_	*F*_*p*,*x*_/*F*_*T*,*x*_
DR	231.78	2900.56	0.01277	−0.00040	46.1	0.065	12.2	1.58	−31.9	0.72	0.28
DF	184.40	2900.56	0.00808	—	—	—	—	—	—	—	—
DFb	179.59	2812.23	0.00816	—	—	—	—	—	—	—	—
ER	192.64	2812.23	0.00938	−0.00195	44.5	0.067	3.5	1.17	−4.8	0.75	0.25
EF	178.15	2812.23	0.00803	—	—	—	—	—	—	—	—
KR	206.49	2812.23	0.01078	−0.00031	47.0	0.062	6.2	1.20	−34.8	0.74	0.26
KF	188.11	2812.23	0.00895	—	—	—	—	—	—	—	—

The smooth wall RANS studies are carried out with a one-dimensional grid containing 143 grid points, stretched in the vertical direction, as per Lloyd *et al.* [40]. Each simulation is labelled with two letters; the first represents the simulation type (*D* for DNS, *E* for EB-SSG, *K* for *k* − *τ*) and the second represents the surface type (R for rough, F for flat). Mesh information for all cases are presented in table 1, with DFb representing the second reference DNS case. Reference values of *u*_*τ*0_ required for calculation of *w*^+^ = *wu*_*τ*0_/*ν* and $C_{\;f0}=u_{\tau 0}^{2}/2U_{b}^{2}$ are taken as corresponding smooth-walled channel flow values for the same *Re*_*b*_. The minor variations in *w*^+^ are due to differences in the treatment of turbulence, where KR slightly overpredicts (flat plate) friction and ER slightly underpredicts (flat plate) friction.

Estimates are made for the virtual origin in table 2, following the recommendation of Chan *et al.* [43]. For low Reynolds number and low blockage ratio flows the virtual origin can have a significant impact on the presentation of turbulent statistics, yet the usual Clauser type method (see e.g. [44]) is inappropriate since the logarithmic region of the boundary layer is poorly defined. Instead, we estimate the virtual origin by assuming the inner-scaled spatially averaged mean flow velocity, $\left\langle {\bar{U}}^{+}\right\rangle$ approaches zero at the inner-scaled virtual origin, $y_{0}^{+}=y_{0}u_{\tau }/\nu$, where *y*_0_ is the virtual origin. Following Chan *et al.* [43] this approach is made more robust by instead assuming the flow velocity evolves linearly with wall distance. We then seek the point where the mean velocity is equal to 1, and then shift the *y*-coordinate by *y*^+^ = −1. All three models predict similar virtual origins, ranging from 0.062 to 0.067. This difference in virtual origin between the three methods is approximately 7% of the maximum denticle height, *h* = 0.072.

Equivalent (dimensionless) sand grain roughness heights ($k_{s}^{+}$) are also estimated in table 2. Here, we follow the method of Boomsma & Sotiropoulos [17] by performing a best fit to the curve

3.1 $${\bar{U}}^{+}=\frac{1}{\kappa }\ln\left(\frac{y-y_{0}}{k_{0}}\right),$$

for $y^{+}=(y-y_{0})u_{\tau }/\nu >50$, derived from the boundary layer log-law, where ${\bar{U}}^{+}$ is the mean velocity (temporally and block-wise averaged for the DNS) scaled by the friction velocity $u_{\tau }\;$, *κ* is the Kármán constant, *y* is the vertical coordinate, *y*_0_ is the virtual origin and *k*_0_ is the roughness height. Note that these estimates should be treated with caution, given their dependence on the log-law formulation. Nevertheless, calculation of $k_{s}^{+}=k_{s}u_{\tau }/\nu$ provides a direct comparison to the simulations of Boomsma & Sotiropoulos [17] who performed DNS over denticles at a similar Reynolds number.

*k*_0_ is obtained by assuming that 1/*κ* = 2.44. The sand-grain roughness, *k*_*s*_ is assumed to be related to the roughness height by *k*_0_ = 0.33 *k*_*s*_, and subsequently scaled by $u_{\tau }$ and *ν* to obtain $k_{s}^{+}$ [27]. Case DR obtains the largest $k_{s}^{+}$ of 12.2, followed by KR predicting $k_{s}^{+}=6.1$, and ER predicting $k_{s}^{+}=3.5$. While DR and KR obtain values in the transitionally smooth regime, case ER could be interpreted as marginally hydraulically smooth. The denticles of Boomsma & Sotiropoulos [17] have an equivalent sand grain roughness height of $k_{s}^{+}=6.2$.

The friction coefficient is calculated by $C_{f}=F_{T,x}/\frac{1}{2}\rho U_{b}^{2}L_{x}L_{z}$, where *F*_*T*,*x*_ is the total streamwise drag force equal to the sum of streamwise viscous (*F*_*ν*,*x*_) and pressure (*F*_*p*,*x*_) forces, calculated by

3.2 $$F_{\nu }=\int_{S}f_{\nu ,i}\partial S\;\mathrm{and}\;F_{p}=\int_{S}f_{\;p,i}\partial S,$$

where *f*_*ν*,*i*_ and *f*_*p*,*i*_ are the local spatially varying viscous and pressure forces on the denticle surface, defined as

3.3 $$f_{\nu ,i}=\nu \frac{\partial {\bar{U}}_{i}}{\partial x_{j}}{\hat{n}}_{j}\;\mathrm{and}\;f_{\;p,i}=(\bar{P}-P_{\infty }){\hat{n}}_{i},$$

where ${\hat{n}}_{j}$ is the local surface-normal vector. We define the reference pressure, *P*_∞_, as the spatially (and temporally for DNS) averaged pressure at *y* = *δ*. Similarly, the net lift coefficient is given by $C_{l}=F_{T,y}/\frac{1}{2}\rho U_{b}^{2}L_{x}L_{z}$. There are vast differences between the drag calculations, relative between rough and smooth surfaces, of the three modelling techniques (table 2). The DNS predicts a drag increase of 58% for the flow over (unribletted) shark skin, in strong agreement with the simulations of Boomsma & Sotiropoulos [17] who predicted an increase of approximately 50% for ribletted mako scales of a similar size. While speculative, it is plausible that the riblets of the Boomsma & Sotiropoulos [17] denticles account for the 8% lower drag, which reduces the equivalent sand grain roughness size (table 2). As we will show, these high drag values are strongly influenced by the low blockage ratios *δ*/*h* of both the present study (*δ*/*h* ≈ 14) and that of Boomsma & Sotiropoulos [17] (*δ*/*h* ≈ 8), explaining their deviation from previous laboratory experiments (e.g. [5,8,18]).

The RANS simulations also predict an increase in friction, but only an increase of approximately 20%. The DNS solution shows that pressure drag accounts for 28% of the total drag force (table 2), which is in close agreement with the 26.35% contribution obtained by Boomsma & Sotiropoulos [17]. Despite the underprediction of *C*_*f*_ by the RANS models (table 2) the contributions to *F*_*T*_ from viscous and pressure forces are well predicted. *F*_*p*_ and $F_{\nu }$ are underpredicted by the RANS simulations, but the distribution of the forces is well captured.

Lift coefficients are also reported in table 2. The DNS case (DR) predicts a weak negative lift coefficient, over an order of magnitude smaller than the drag coefficient. This is well replicated by the *k* − *τ* model, but the EB-SSG model predicts a much stronger downward lift force, approximately six times stronger than the *k* − *τ* and DNS solutions.

**Figure 4.**
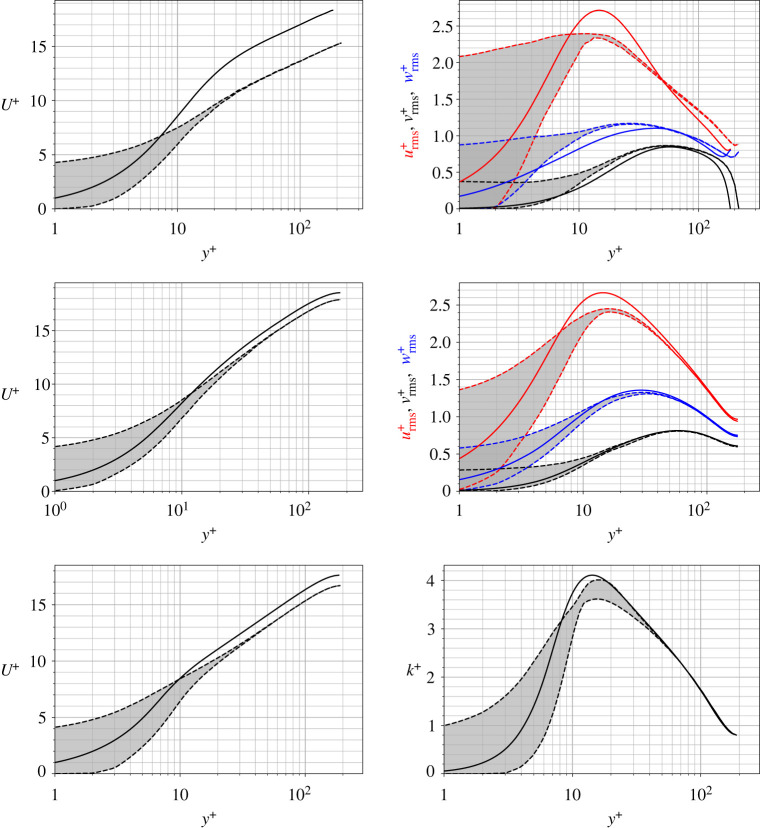
Profiles of mean streamwise velocity (left) and RMS velocities (right). Means for the DNS are calculated using temporal and block-wise averaging. Data from top to bottom are DNS, EB-SSG and *k* − *τ*. Note that *k*^+^ is plotted for *k* − *τ* rather than individual components of the Reynolds stresses. The rough-wall datasets have their wall-normal coordinate with its origin at the virtual origin (table 2). Rough-wall datasets are plotted with dashed lines representing the bounds of their values (which vary in space due to the roughness), while flat-wall data are plotted in solid lines.

Profiles of inner-scaled (scaled by respective friction velocities, $u_{\tau }$) mean velocity and TKE are plotted in figure 4. Block-wise spatial and temporal averaging is performed for the DNS solutions, preserving the spatial variations of means and RMS statistics over the denticles (i.e block-wise spatial averages are obtained by collapsing the 24 × 12 denticle domain to a single denticle section, equal to the RANS domain size). Note that the RANS solutions do not require averaging, since the steady-state turbulent statistics are directly solved. For the rough surfaces the bounds of respective variables are shaded in grey rather than performing a spatial mean. These bounds do not necessarily correspond to distinct vertical profiles above the rough surface. In addition, the wall-normal distance is scaled by $u_{\tau }/\nu$ with the origin placed at the virtual origin (table 2), such that $y^{+}=(y-y_{0})u_{\tau }/\nu$. Each rough-wall dataset is plotted alongside the corresponding flat-wall dataset calculated with the same turbulence closure. Direct comparisons between the different methodologies are not useful due to differences in the flat-wall predictions (see e.g. [40,45]); the relative differences between flat-wall and rough-wall predictions are more informative here, where we can assess if RANS models capture the correct trends in mean flow statistics. There are clear similarities between the three modelling methods when assessing the mean velocity profiles of figure 4. The rough surface leads to a downward offset of the logarithmic and wake regions of the boundary layer ($y^{+}≳30$) when compared to the smooth wall simulations, and all three methods obtain similar bounds of mean velocity which converge to a homogeneous profile for $y^{+}≳30$. Differences only lie in the magnitude of the downwards offset, arising due to the differences in $u_{\tau }$, where the rough-wall DNS predicts a much larger offset from the smooth wall solution than the RANS models.

TKE, *k*^+^, is also plotted in figure 4 for each dataset, with individual components plotted for the DNS and EB-SSG (e.g. ${\left(u_{\mathrm{rms}}^{+}\right)}^{2}={\bar{uu}}^{+}$) cases (noting that *k* − *τ* directly solves for *k* rather than individual components of the Reynolds stress tensor). The EB-SSG and DNS models are in reasonable agreement, particularly with the streamwise velocity fluctuations, $u_{\mathrm{rms}}^{+}$. Also, the EB-SSG and DNS solutions converge to a spatially uniform solution near the peak value of TKE, while the *k* − *τ* solution still has quite a wide spread of values at *y*^+^ ≈ 15. However, disagreement with DNS is more apparent when observing the vertical (*v*) and spanwise (*w*) components of TKE, where the DNS predicts an increase in magnitude while EB-SSG predicts a slight decrease in magnitude of $w_{\mathrm{rms}}^{+}$. The increase in $v_{\mathrm{rms}}^{+}$ and $w_{\mathrm{rms}}^{+}$ with a decrease in $u_{\mathrm{rms}}^{+}$, obtained with the DNS, indicates a more isotropic flow than when roughness is not present, which is not as well captured by the EB-SSG model. However, trends are reasonably well predicted by the two RANS models, despite the underprediction of the drag coefficient.

**Figure 5.**
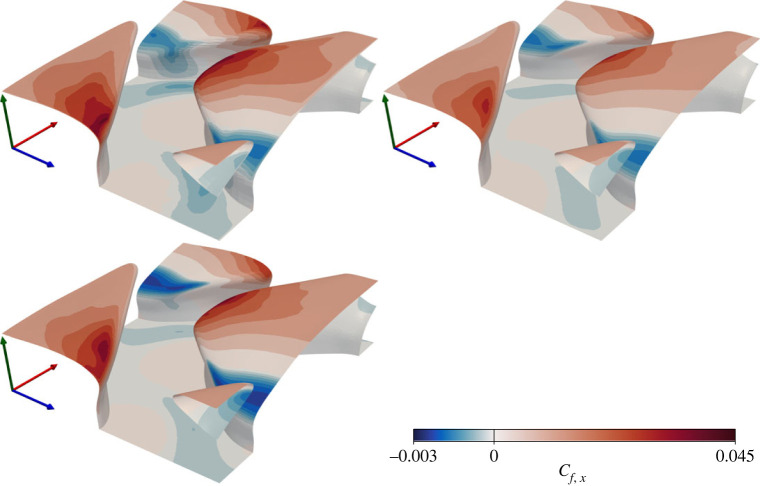
Local streamwise friction coefficient for DR (upper left), ER (upper right) and KR (lower left) solutions. Note the asymmetric scaling of the colourbar about *c*_*f*,*x*_ = 0 to highlight recirculation regions. Axes are coloured by *x* (red), *y* (green), *z* (blue).

The spatially varying streamwise coefficient of friction, $c_{\;f,x}=f_{\nu ,x}/\frac{1}{2}\rho U_{b}^{2}$, is plotted over the shark scale surface in figure 5. The colourbar has been scaled asymmetrically about *c*_*f*,*x*_ = 0 to highlight regions of negative friction coefficient (i.e regions with reverse flow). All three models lead to near identical solutions for the friction distribution over the surface, with a local peak on the upper surface of the denticle crown. The peak is off-centre, due to shielding from the upstream denticle, such that the outer edge is more exposed to high-speed fluid. The upstream denticle also causes regions of recirculation at the front of the denticle crowns. The regions between denticles have very low levels of friction where the rough surface provides protection from high-speed fluid. Predictions of the different models only differ in their magnitude, although these differences appear minor. The DNS leads to higher friction over the full denticle crown than the other two models, although the regions of high and low friction are well predicted by the RANS models. The recirculation zone is well captured by EB-SSG when compared to the DNS, but the *k* − *τ* model predicts a much higher (negative) peak in friction coefficient.

**Figure 6.**
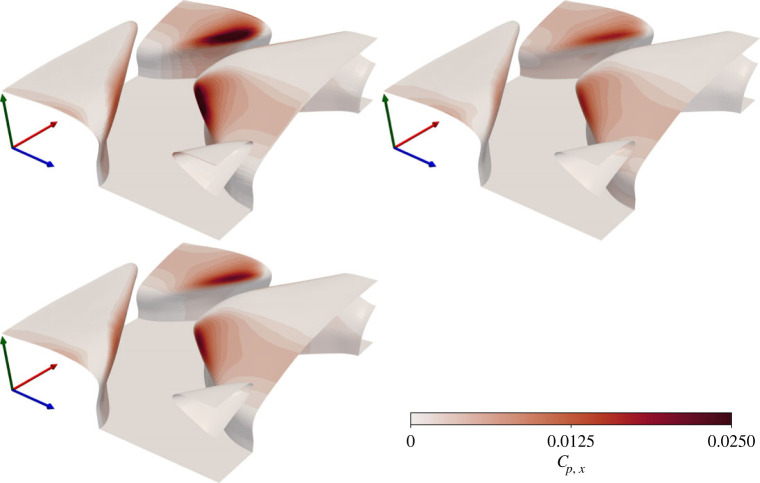
Local streamwise pressure coefficient for DR (upper left), ER (upper right) and KR (lower left) solutions. Axes are coloured by *x* (red), *y* (green), *z* (blue).

Plots of the local streamwise pressure coefficient, $c_{p,x}=f_{\;p,x}/\frac{1}{2}\rho U_{b}^{2}$, are consistent with those of friction, observed in figure 6. All three simulations lead to very similar pressure distributions, differing only in their magnitude. Pressure distributions peak at the upstream edge of the denticle crown, off-centre where the denticle is most exposed. Pressure drag is negligible over most of the denticle, except at the region most exposed where there is a strong peak. The DNS leads to a stronger peak in *c*_*p*,*x*_ than the RANS models, although the contour values over the rest of the denticle surface are in good agreement. The underprediction of *C*_*f*_ associated with the RANS models (table 2) is clearly a result of the underprediction of the peak values of *c*_*p*,*x*_ at the exposed edge of the denticle, and the slight underprediction of *c*_*f*,*x*_ over the full denticle crown. When integrated over the full surface these slight differences in local drag sum to the discrepancies in *C*_*f*_ (table 2). Despite this, the general trends are well predicted by the RANS models.

**Figure 7.**
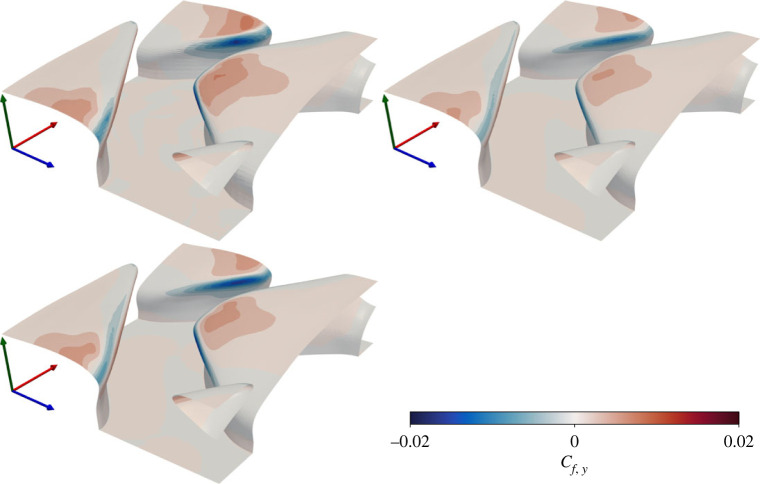
Local vertical friction coefficient for DR (upper left), ER (upper right) and KR (lower left) solutions. Axes are coloured by *x* (red), *y* (green), *z* (blue).

**Figure 8.**
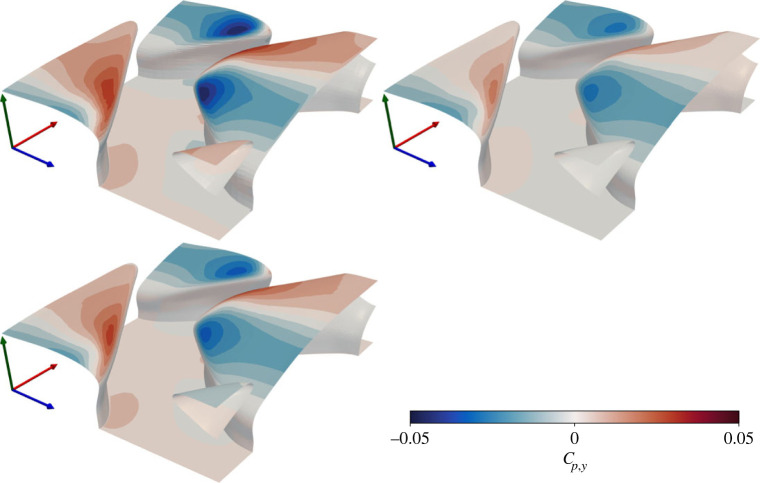
Local vertical pressure coefficient for DR (upper left), ER (upper right) and KR (lower left) solutions. Axes are coloured by *x* (red), *y* (green), *z* (blue).

Contours of vertical pressure and friction coefficients (lift), based upon the vertical friction (*f*_*ν*,*y*_) and pressure (*f*_*p*,*y*_) forces, are presented in Figures 7 and 8. Friction forces lead to local upward lift over the denticle crown, and downward lift at the denticle edges. Pressure forces result in a downward lift force on the denticle leading edge, and upward lift at the downstream edge. Upward/downward forces approximately balance for all three cases, leading to a net weak downward lift coefficient an order of magnitude weaker than the streamwise drag coefficient (table 2). Like the streamwise coefficients, the vertical coefficients are qualitatively similar for all three simulations. While qualitatively similar, EB-SSG leads to overall weaker downward pressure forces than the *k* − *τ* and DNS solutions, primarily due to a weaker pressure force acting on the leading edge of the denticle crowns. Interestingly the maximum/minimum values of the lift coefficients are greater than those of the drag coefficients. Further, when observing the vertical pressure coefficient there is a clear moment acting on the denticle in the *z*-axis, with downward pressure forces acting on the front of the denticle crown, and upward pressure forces acting on the back of the denticle crown. If the denticle were not fixed at the base, this moment would enable the denticle to bristle, conceivably similar to the bristling dynamics observed experimentally (e.g. [10]).

**Figure 9.**
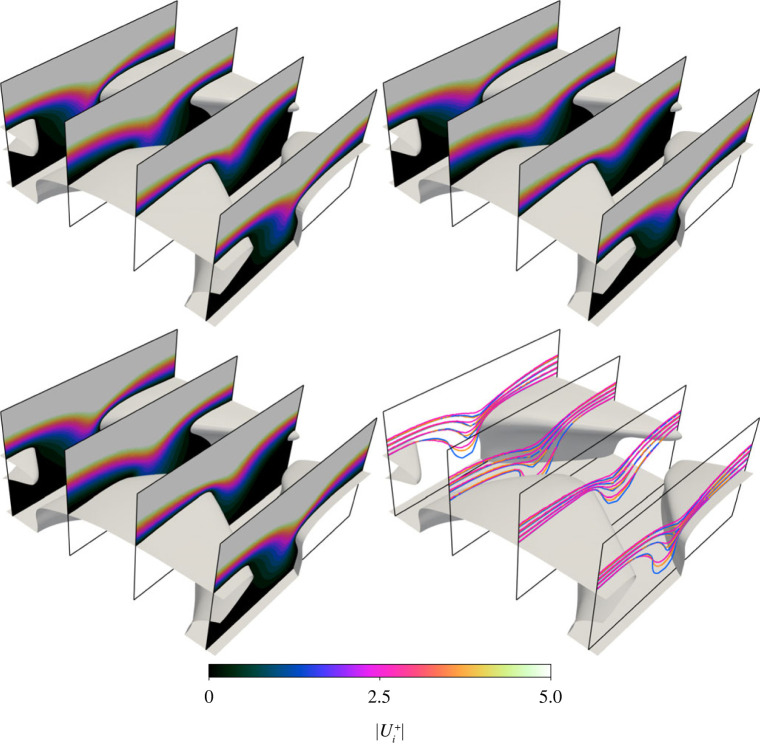
Contours of velocity magnitude on four *x*-normal slices through the domain, near the denticle surface. Flow direction is approximately from upper left to lower right. Contour data with corresponding colourbar are DR (upper left), ER (upper right) and KT (lower left). The lower right sub-figure combines all three datasets with contour values of $|U_{i}^{+}|=1,2,3,4$. Colours represent DR (blue), ER (pink) and KR (orange).

Contours of velocity magnitude are plotted on slices through the domain near the denticle surface in Figure 9. Here the velocity magnitudes are normalized by respective friction velocities. Normalized in this way we see little difference between the three simulations. Figure 9 reveals relatively high-speed fluid between the denticles, with high gradients at the exposed edges of the denticle crown. This could be considered a high momentum pathway where relatively high-speed fluid passes between denticles and is responsible for sustaining the back-flow regions behind denticles and increased pressure drag at their exposed edges. This high velocity is the cause of the locally high pressure and friction coefficients, where the denticles are not adequately shielded by the denticles upstream. Differences between the three datasets are more clear when contours are plotted together (lower right of figure 9). Differences arise only in the region between denticles, where DR solutions have a consistently higher flow velocity, followed by KR and then ER data.

**Figure 10.**
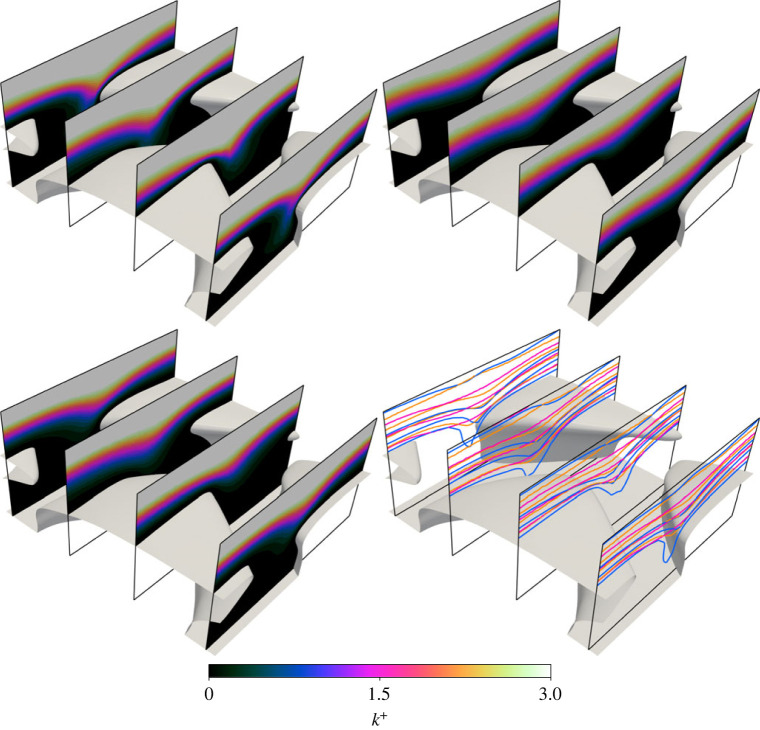
Contours of TKE on four *x*-normal slices through the domain, near the denticle surface. Flow direction is approximately from upper left to lower right. Contour data with corresponding colourbar are DR (upper left), ER (upper right) and KT (lower left). The lower right sub-figure combines all three datasets with contour values of *k*^+^ = 0.5, 1.5, 2.5, 3.5. Colours represent DR (blue), ER (pink) and KR (orange).

Differences between the datasets are more clear when visualizing the TKE, as per figure 10. All three models predict that TKE has its maximum value above the surface (above the contour range), and that TKE is negligible beneath the denticle crowns. However, the RANS models (ER and KR) both predict a lower TKE in the region between denticles, when compared to the DNS. This is most clear for the *k*^+^ = 0.5 contour which penetrates much deeper beneath the denticle crown for the DNS. The *k* − *τ* model leads to a very similar TKE field as EB-SSG, although there is evidence that TKE is marginally higher for *k* − *τ* predictions between the denticles. The discrepancy in TKE data may explain the poor agreement between DNS and RANS models for total drag increase (table 2); fully resolving the flow leads to a high peak in TKE near and between the denticle surface which acts as a momentum sink and increases mixing, subsequently increasing the fluid velocity near the denticles and increasing both pressure and viscous drag.

**Figure 11.**
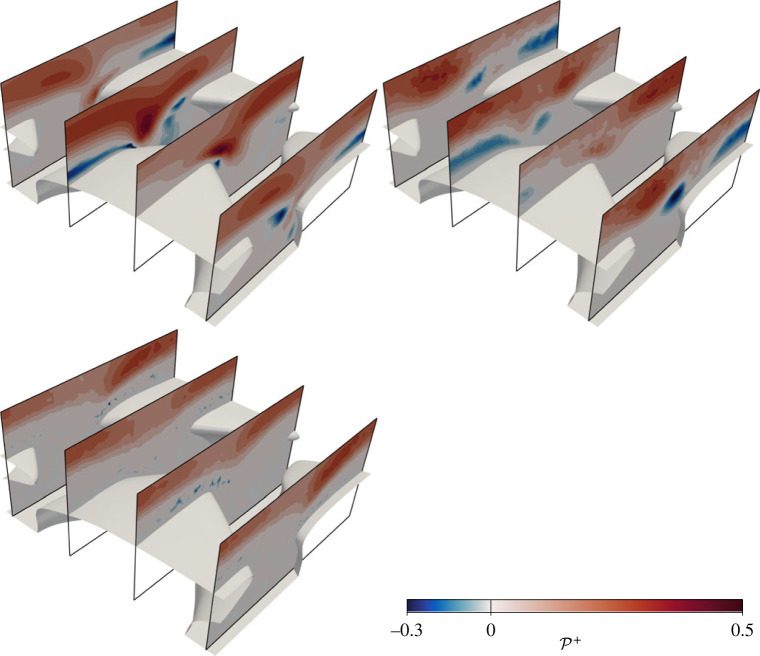
Contours of TKE production on four *x*-normal slices through the domain, near the denticle surface. Flow direction is approximately from upper left to lower right. Contour data with corresponding colourbar are DR (upper left), ER (upper right) and KT (lower left).

The production of TKE, $P^{+}=-{\bar{u_{i}u_{j}}}^{+}\partial_{j}{\bar{U}}_{i}^{+}$, is shown in figure 11 for the three denticle simulations. Here differences between the datasets are more pronounced, particularly in the region between denticles. The DNS (DR) predicts a peak production of TKE in the region between denticles, where they are most exposed to high-speed fluid. There is also a peak in $P^{+}$ close to the edge of the denticle crown for case DR, evidenced at the most upstream slice in figure 11. Regions of negative production are also present, particularly in regions of reverse flow (corresponding to negative *c*_*f*_ in figure 5). Neither ER or KR data show the large peak in TKE production when compared to the DNS, but the EB-SSG model does provide reasonable predictions of the negative production. Increased turbulence, and therefore drag, for case DR is, therefore, associated with the increased production of TKE near and between the denticles which the two RANS models are unable to reproduce.

**Figure 12.**
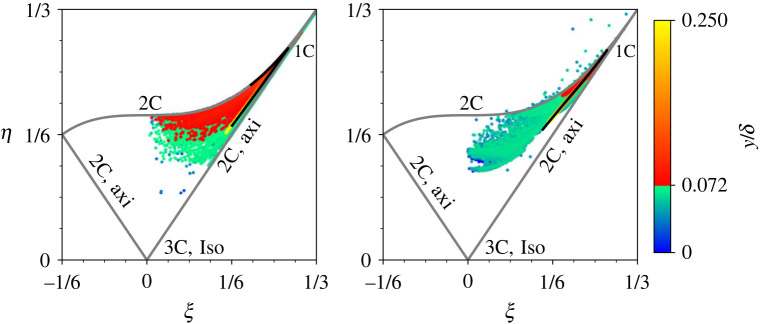
Anisotropy of near-wall (*y* ≤ 0.25*δ*) turbulence as quantified by the Lumley triangle for DNS data (left) and EB-SSG data (right). Flat plate data (DF,EF) are presented by solid black lines and rough-wall data (DR,ER) are plotted with a colourmap indicating their vertical coordinate. The colourbar has been truncated at *y* = 0.072*δ* which is the maximum denticle height. The bound labels of the Lumley triangle represent one component (1C), two component (2C), axisymmetric two component (2C, axi) and isotropic three-component (3C) turbulence.

The increased turbulence from roughness leads to more isotropic turbulence near the wall, evidenced by plotting DNS data on the Lumley triangle (figure 12). Here the isotropy is quantified by calculation of *η* and *ζ* which are related to the second and third invariants of the Reynolds stress anisotropy tensor, $b_{ij}=\bar{u_{i}u_{j}}/2k-\frac{1}{3}\delta_{ij}$. *η* and *ζ* relate to *b*_*ij*_ by

**Figure 13.**
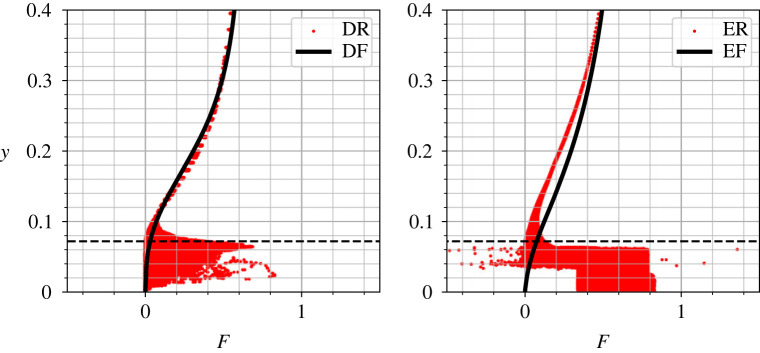
Vertical dependence of the anisotropy function *F*, for cases DR and DF (left), and ER and EF (right). The dashed line represents the maximum denticle height, *h* = 0.072.

3.4 $$6\eta^{2}=-2{\mathrm{II}}_{b},\;\text{and}\;6\zeta^{3}=3{\mathrm{III}}_{b},$$

where II_*b*_ = −*b*_*ij*_*b*_*ji*_/2 and III_*b*_ = *b*_*ij*_*b*_*jk*_*b*_*ki*_/3 are the second and third invariants of *b*_*ij*_. The coordinate system (*ζ*, *η*) is a useful tool to assess the isotropy of turbulence, and identifies whether there are dominant eigenvalues of *b*_*ij*_ [46]. The DNS flat plate solution indicates primarily two-component axisymmetric flow, transitioning to two-component flow in the very near-wall region [46], which is reasonably well predicted by EB-SSG. Differences between rough and smooth surfaces are much more clear. Here, each data points represents a GLL point (DR) or cell centre value (ER), coloured by its *y* coordinate, with a colourmap truncated at the maximum denticle height to approximately identify regions between and beneath denticles, and regions above them. There is a clear large spread in data, where the rough surface deviates considerably from the flat plate solution near and beneath the denticle. We see a broad range of turbulent structure, spanning two-component flow through to axisymmetric flow, and then convergence to the flat plate solution further from the denticle surface. There is some agreement between ER and DR cases, although ER does not predict quite as large a spread in the anisotropy data near the denticle surface. This is more obvious when plotting the vertical variation of the anisotropy function,

3.5 $$F=1+27{\mathrm{III}}_{b}+9{\mathrm{II}}_{b},$$

as per figure 13, where *F* is bounded between zero and one, vanishing for two-component turbulence and approaching unity for isotropic 3C turbulence [47]. Both simulations DR and ER approach a spanwise homogeneous state for y≳0.1, but the DNS (DR) shows a wider spread of data near the denticle surface, *y* = 0.072. This is particularly notable for the points near y ≈ 0.072δ, where DR data show substantially larger deviation from the flat plate solution. ER data begin to collapse onto the flat plate solution at a smaller *y* value when compared to DNS, suggesting that the blending functions require some adjustment for accurate rough-wall predictions. Further, there are a few data points that lie outside the Lumley triangle, and the theoretical bounds of the anisotropy function, indicating that realisability conditions are not fully met for case ER. These issues perhaps explain why the EB-SSG model underpredicts production of near-wall turbulence and therefore drag.

**Figure 14.**
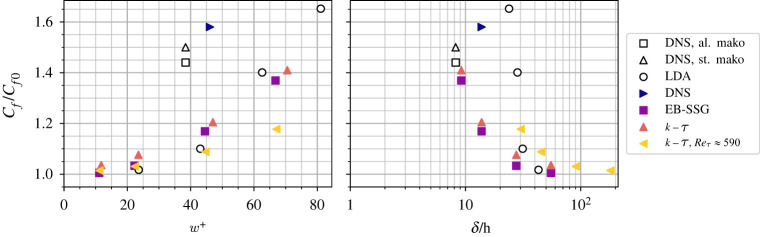
Relative drag coefficient predictions for DNS and RANS models, compared to the LDA data of Lloyd *et al.* [18] for the same denticle geometry, and the ribletted mako denticle DNS of Boomsma & Sotiropoulos [17], with ‘al.’ referring to their case with aligned denticles, and ‘st.’ referring to their case with staggered denticles. All datasets are for $Re_{\tau }\approx 180$ except one additional *k* − *τ* case carried out at $Re_{\tau }\approx 590$, and the LDA data where $Re_{\tau }$ varied between approximately 299 and 728. Relative drag is plotted as a function of dimensionless denticle width, *w*^+^ (left), and blockage ratio, *δ*/*h* (right), where *h* is taken as the maximum denticle height, and *δ* represents either the channel height or the boundary layer height for the LDA data of Lloyd *et al.* [18].

However, while improvements could be made to the RANS models to better predict near-denticle turbulence, comparisons to DNS show that the flow is qualitatively well predicted, particularly the distribution of pressure and viscous forces over the denticle surface. The ER and KR cases have, therefore, been repeated for three additional *w*^+^ values to make comparisons to the experiments of Lloyd *et al.* [18]. In addition, the *k* − *τ* simulations have been repeated for *Re*_*b*_ = 10865 ($Re_{\tau }\approx 590$) to assess the influence of the blockage ratio, *δ*/*h*, on drag predictions, where *h* is the maximum denticle height. The drag predictions relative to flat plate solutions are presented in figure 14 as a function of dimensionless denticle width *w*^+^, and blockage ratio *δ*/*h*. Very close agreement is observed between the two RANS models, EB-SSG and *k* − *τ*. Both models show increased drag as *w*^+^ increases, and convergence to *C*_*f*_/*C*_*f*0_ → 0 as *w*^+^ → 0 (i.e roughness has a negligible effect on drag when it is vanishingly small). The present DNS is in reasonable agreement with the DNS of Boomsma & Sotiropoulos [17], with the narrower (smaller *w*^+^) ribletted denticles of Boomsma & Sotiropoulos [17] leading to a relative drag coefficient 8% lower than the smooth denticles herein, and 14% lower when denticles are aligned rather than staggered (thus better shielding downstream denticles from high momentum fluid). The RANS models appear to agree well with the experimental laser Doppler anemometry (LDA) data of Lloyd *et al.* [18], although this agreement should be treated with caution, noting that the LDA data were collected for a developing boundary layer over $Re_{\tau }\approx 299$–728, with moderate freestream turbulence. RANS data can, therefore, only be directly compared to the DNS data at *w*^+^ ≈ 45. Despite this, the trends appear well captured by the RANS models when compared to the laboratory experiments.

The influence of $Re_{\tau }$ on drag is demonstrated in figure 14 by repeating $Re_{\tau }\approx 180$ simulations at $Re_{\tau }\approx 590$ using the *k* − *τ* model at the same denticle widths (*w*^+^). A clear reduction in drag is observed when $Re_{\tau }$ (and therefore *δ*/*h*) is increased, most pronounced at higher *w*^+^, where a 20% lower drag is obtained at *w*^+^ ≈ 70. This effect arises, at least in part, due to blockage effects where denticles extend through a larger portion of the boundary layer at low $Re_{\tau }$. As a consequence, the impact of roughness can be felt over the full boundary layer height, rather than being limited to the inner region (y≲0.2δ) of the boundary layer [27,48,49]. For a boundary layer flow to be free from blockage effects Jiménez [27] and Flack *et al.* [48] suggest a blockage ratio of *δ*/*k*_0_ > 40 is required, where *k*_0_ is the roughness height. At *w*^+^ ≈ 70 the Reynolds number increase from $Re_{\tau }=180$ to 590 relates to an increase in blockage ratio from *δ*/*h* ≈ 10 to 30, explaining the reduced drag obtained by the *k* − *τ* simulations of figure 14.

There is a large range of blockage ratios presented in figure 14, many data points have *δ*/*h* < 40, and as low as *δ*/*h* ≈ 8 for the DNS of Boomsma & Sotiropoulos [17] (although it should be noted that, while more difficult to calculate and often not reported, an average denticle height may be more appropriate than the maximum denticle height *h* when considering blockage ratios). This is considerably lower than 40, providing a reasonable explanation as to the high drag predictions by low Reynolds number DNS. It should also be noted that the LDA data are also affected by blockage, albeit less-so than the DNS and RANS simulations at $Re_{\tau }=180$. This suggests that the higher *w*^+^ measurements of Lloyd *et al.* [18] may be influenced by blockage, and therefore increased drag. However, this scrutiny cannot be readily applied to other experimental measurements of boundary layers over shark scales, given only Lloyd *et al.* [18] has obtained the required velocity profiles, and therefore boundary layer thickness, *δ*.

## 4. Discussion and conclusion

Flows over shark skin surfaces are complex and poorly understood. While there has been much research quantifying flows over simpler surfaces (e.g. riblets), there is a dearth of research investigating the fluid dynamics of boundary layer flows over more intricate and three-dimensional denticles. There is evidence that the skin friction drag reduction of these surfaces may be at least as efficient as riblets, if not more-so (see e.g. [5,7,8,19]). However, DNSs of such flows (presented herein and those of Boomsma & Sotiropoulos [17]) predict a greatly enhanced skin friction relative to smooth surfaces, appearing at odds with experimental work. Consolidation of these two bodies of work would be of great benefit to the scientific and engineering communities, enabling questions to be answered regarding the evolution of shark skin, and the development of novel drag reducing engineering surfaces.

There are two key contributing factors to our poor understanding of these flows, and the conflicting data reported by previous studies (as discussed in §1). Firstly, denticle geometries vary considerably between different experiments, arising naturally due to differences in shark species and location on the shark body, and also due to differences in manufacturing techniques where, for example, moulding methodologies can obtain highly intricate shark skin surfaces but cannot replicate the cavities between and underneath overlapping denticles [19]. Secondly, the majority of previous studies have focused on the use of force balances to measure drag imposed by denticles; only a handful of studies have attempted to measure/predict near-denticle (boundary layer) flow fields [17,18]. Yet some of the most informative comparative studies on longitudinal riblets have obtained these flow fields (e.g. [11–13]); without quantification of these flow dynamics we cannot hope to understand how shark skin flows differ from other rough surfaces, or how riblets interact in combination with shark skin denticles.

There are clear challenges with obtaining flow field information close to rough surfaces in the laboratory. While optical techniques may seem promising, they are unable to obtain the very-near-denticle flow fields, when noting that the most important (drag reducing) fluid dynamics occur when denticles (or riblets) have length scales of order $\delta_{\nu }=\nu /u_{\tau }$. The numerical methodologies suggested in this paper, SSEM DNS and RANS, offer some clear advantages over laboratory experiments in understanding the small scale flow dynamics that underpin the drag characteristics of flow over shark scales. SSEM is a high-fidelity numerical technique capable of obtaining fully resolved flow fields over complex denticle geometries at spectral accuracy using body-fitted elements. This paper has demonstrated its capabilities by resolving a periodic open-channel flow with smooth shark scales on the wall, obtaining results in good agreement with the previous simulations of Boomsma & Sotiropoulos [17]. A drag increase of 58% is obtained, 8% higher than that of the ribletted denticles of Boomsma & Sotiropoulos [17] at a similar denticle width. This difference in drag may be due to the riblets on the denticles of Boomsma & Sotiropoulos [17], although there are other differences in geometry which also likely influences drag. For example, the denticles herein are shorter and wider than the mako denticles of Boomsma & Sotiropoulos [17]. Estimations of the equivalent sand-grain roughness height show that the smooth denticles have a sand-grain roughness height approximately twice that of the denticles investigated by Boomsma & Sotiropoulos [17]. While roughness is still transitional, this increase in roughness size leads to the higher drag.

However, it is important to note that the present DNS study, and indeed the study of Boomsma & Sotiropoulos [17], appear at odds with other literature investigating shark skin performance in boundary layer flows. Yet these discrepancies can be explained when considering the flow blockage ratios, *δ*/*k*_0_, which must be considered when assessing flows over rough surfaces. Jiménez [27] suggests a blockage ratio of *δ*/*k*_0_ > 40 is required for roughness effects to be limited to the near-wall flow (*y*/*δ* < 0.2), yet the DNS presented here, and that of Boomsma & Sotiropoulos [17], have *δ*/*h* < 15. The impact of blockage ratio on denticle drag is well demonstrated in figure 14, where RANS methodology has been used to scale up simulations to higher Reynolds numbers, and subsequently higher blockage ratios. By increasing the blockage ratio to 45, the drag increase due to the denticles is halved from approximately 20–10%, for the same size denticles (*w*^+^ ≈ 45). Blockage ratios may, therefore, help explain some of the discrepancies between previous studies, where experiments and realistic flows over shark scales will likely have much higher blockage ratios.

Despite blockage ratio limitations, our DNS has shed light onto the drag and lift generation mechanisms present on the flow over smooth shark scales. The SSEM-DNS indicates that 28% of the total drag is associated with pressure drag acting on the exposed edge of the denticle crowns, due to the lack of shielding from the upstream denticles. Both viscous and pressure drag peak at the edge of the denticle crown, arising due to the emergence of a high momentum pathway between individual denticles, drawing in relatively high-speed fluid which impinges on the denticles. As high-speed fluid is drawn between the denticles we observe flow separation at the trailing edges of the denticle crowns, and an increase in shear and TKE production. Drag is expected to be reduced considerably if denticles were more tightly packed (noting, however, that the denticle spacings were chosen to match those of Lloyd *et al.* [18], which were determined by three-dimensional printing capabilities, and that there are many shark species that do have sparse denticle layouts, as shown by Reif [4]).

We further observe a positive spanwise torque acting on the denticle, primarily due to the high magnitude pressure forces acting on the denticle crown, with an upward pressure force acting at the back of the denticle crown, and a negative pressure force acting on the front of the denticle crown. The observed torque may explain the bristling observed in experimental studies [10]. Further work should pursue the time-dependence of these forces, particularly for movable denticles, given that bristling is a time-dependent process. However, it is interesting to speculate that a bristled shark scale may well reduce the high magnitude pressure forces acting on the downstream denticle (e.g. figure 6) due to increased shielding, therefore, reducing the total friction coefficient.

However, while DNS using SSEM offers a feasible methodology to obtain highly accurate flow field data, the technique is limited to low Reynolds numbers, and requires extensive computational cost to resolve flow dynamics (even when accounting for the cost savings introduced by overlapping spectral element meshes). The SSEM could be readily extended to include sub-grid scale closures (large eddy simulations) but cost savings would not be appreciable given that small near-denticle elements are required to resolve surface curvature. The limitation of DNS to low Reynolds numbers (and subsequently low blockage ratios, *δ*/*h*) makes RANS methodology a more tractable alternative, particularly concerning periodic flows where the fluid domain can be constructed to contain just a single denticle. This study has demonstrated that both complex eight-equation Reynolds stress closures (EB-SSG) and two-equation eddy-viscosity models (*k* − *τ*) are capable of obtaining very similar flow characteristics to DNS. Both RANS models are able to predict the correct contributions of viscous and pressure forces to total drag and how these forces are distributed over the denticle surface, and they also obtain correct trends in velocity and TKE data. However, current RANS models underpredict near-wall production of TKE induced by the rough surface. As a consequence, the drag increase predicted by RANS models is only 20%, compared to the 58% obtained by DNS. A parameter study on the effect of *w*^+^ on drag (figure 14) indicates that the correct trends are obtained when compared to both DNS and experimental results. While there is promise in RANS methods, future work should investigate modification to the models to calibrate for the increased production of TKE near the wall, and how these properties change with denticle geometry.

Laboratory experiments, RANS simulations and DNS are all important methodologies for understanding how shark scale geometry influences drag. Laboratory techniques are able to simulate flows over realistic shark skin geometries, subject to fluid flows free from numerical modelling (e.g. turbulence closure) errors. However, even with current state-of-the-art optical measurement techniques the near- and between-denticle flow fields cannot be captured, due to the small length scales associated with denticles. While denticles could be scaled up in size, this comes with limitations on the potential dimensionless denticle widths, *w*^+^, and blockage ratios, *δ*/*h*, that can be modelled. The ideal laboratory experiments will manufacture denticles small enough to enable measurements at low *w*^+^ (where riblets are known to reduce drag) and high *δ*/*h* (where blockage effects are negligible), but large enough to enable resolution of near-denticle flow with, for example, particle image velocimetry.

SSEM-DNS has a clear advantage over experiments for resolving near- and between-denticle flow dynamics, and is free from modelling errors associated with turbulence closure. However, the limitation of DNS to low Reynolds numbers, and hence low *δ*/*h* for a given *w*^+^, means that high-accuracy simulations will always be somewhat influenced by blockage effects. While DNS is an important tool for fully understanding the fluid dynamics of flows past denticles, it must be complemented by wider parameter studies performed by other techniques.

RANS methods have the potential to bridge the gap between laboratory experiments and DNS by their ability to resolve near-denticle fluid dynamics at higher Reynolds numbers, and therefore higher blockage ratios, *δ*/*h*. This paper has demonstrated their capability to obtain the correct contributions and spatial distribution of viscous and pressure drag, and the correct trends in velocity, TKE and total drag. However, they are currently ill-equipped to account for the increased TKE production near the denticles, likely due to the imposed damping/blending functions. Future adjustments to these models could enable a wide parameter study of shark skin denticle geometry and associated impact on boundary layer flow dynamics.

## Data Availability

Our data are available from the Dryad Digital Respository: https://doi.org/10.5061/dryad.cnp5hqc7z [50].

## Footnotes

1 A more appropriate parameter may be their groove-area *A*_*h*_, discussed by Garcia-Mayoral & Jimenez [15], although *s* is more commonly adopted.
